# The anti-malarial drug atovaquone potentiates platinum-mediated cancer cell death by increasing oxidative stress

**DOI:** 10.1038/s41420-020-00343-6

**Published:** 2020-10-27

**Authors:** James T. T. Coates, Gonzalo Rodriguez-Berriguete, Rathi Puliyadi, Thomas Ashton, Remko Prevo, Archie Wing, Giovanna Granata, Giacomo Pirovano, Gillies W. McKenna, Geoff S. Higgins

**Affiliations:** Department of Oncology, University of Oxford, Oxford, UK

**Keywords:** Chemotherapy, Preclinical research

## Abstract

Platinum chemotherapies are highly effective cytotoxic agents but often induce resistance when used as monotherapies. Combinatorial strategies limit this risk and provide effective treatment options for many cancers. Here, we repurpose atovaquone (ATQ), a well-tolerated & FDA-approved anti-malarial agent by demonstrating that it potentiates cancer cell death of a subset of platinums. We show that ATQ in combination with carboplatin or cisplatin induces striking and repeatable concentration- and time-dependent cell death sensitization in vitro across a variety of cancer cell lines. ATQ induces mitochondrial reactive oxygen species (mROS), depleting intracellular glutathione (GSH) pools in a concentration-dependent manner. The superoxide dismutase mimetic MnTBAP rescues ATQ-induced mROS production and pre-loading cells with the GSH prodrug N-acetyl cysteine (NAC) abrogates the sensitization. Together, these findings implicate ATQ-induced oxidative stress as key mediator of the sensitizing effect. At physiologically achievable concentrations, ATQ and carboplatin furthermore synergistically delay the growth of three-dimensional avascular spheroids. Clinically, ATQ is a safe and specific inhibitor of the electron transport chain (ETC) and is concurrently being repurposed as a candidate tumor hypoxia modifier. Together, these findings suggest that ATQ is deserving of further study as a candidate platinum sensitizing agent.

## Introduction

Platinum-based anti-cancer agents are one of the most common classes of chemotherapeutics in use^1^. Despite initial effectiveness, however, cancer cells eventually elicit an adaptive response, limiting their use as monotherapies^2,3^. As a result, shortly after their discovery, platinums were explored in combination with other non-platinum therapies to mitigate this issue and maximize benefit^4^—a trend which continues today^5–7^. Multi-agent platinum-based treatment regimens are now amongst the most frequently used first- and second-line strategies for a wide-spectrum of cancers^8,9^. Novel and effective platinum-based combinations could therefore continue to have direct and expansive clinical relevance to a large number of patients by providing alternative treatment options. De novo drug discovery efforts are however challenging and complex and so alternative strategies to identify suitable agents are needed. Repurposing of already-approved drugs may bridge this gap while taking advantage of established safety profiles and a priori mechanistic insight, which, together, could facilitate quicker translation into clinical testing.

Cisplatin, the archetypical platinum agent, was first synthesized in 1844 and repurposed for oncology in 1965^10^. It’s actions on mammalian cells are pleiotropic but it principally exerts an anti-cancer effect by inducing irreparable DNA cross-links, preferentially targeting rapidly dividing cells and inducing apoptosis^10,11^. There now exist a plethora of platinum derivatives; however, despite similar structures, their mechanisms of action vary—cytotoxicity of cisplatin and second-generation carboplatin are mediated by nuclear effects^12,13^ while the cell death caused by oxaliplatin, a third-generation platinum with a substituted diaminocyclohexane ligand, has been attributed principally to ribosomal damage^14^. Cisplatin and carboplatin have furthermore been shown to induce a mitochondria-dependent, reactive oxygen species (ROS)-mediated cellular response that can contribute to their cytotoxic effects^15–17^. In this context, mitochondrial ROS (mROS) production is independent of platinum-induced nuclear DNA damage and produced as a consequence of impaired metabolism or mitochondrial protein synthesis. Supporting this theory, exogenous replenishment of thiol group (-SH) antioxidant pools, such as glutathione (GSH), mitigates many of the cytotoxic effects of cisplatin and carboplatin on human cancer cells^18^. Moreover, adaptive endogenous increases in GSH by cancer cells have been demonstrated to confer protection^3^. Together, these reports directly implicate oxidative stress, mROS, and antioxidant activity as key mediators of platinum agent efficacy. Given these links, we hypothesized that pharmacologically increasing mROS could potentiate the anti-cancer effectiveness of both carboplatin and cisplatin.

Previous studies reported preclinically that the well-tolerated anti-malarial drug atovaquone (ATQ) increases ROS production in cancer cells^19–21^ and may overcome acquired platinum resistance in vitro^22^, but any link with platinum sensitization has not yet been investigated. As an anti-malarial in the clinic, ATQ has an excellent safety profile with only mild side effects, can accumulate in tissues, undergoes biliary excretion after 50–84 h, and is not metabolized to a significant extent^23^. Crystallographic studies in mammalian cells demonstrate that ATQ binds directly to and inhibits Complex III (the transmembrane cytochrome *bc*_1_ complex) of the electron transport chain (ETC), suppressing oxidative phosphorylation (OXPHOS) and inducing a metabolic shift towards a more glycolytic phenotype^24–26^. Indeed, loss of function mutations in cytochrome *bc*_1_ have been demonstrated to induce constitutive production of ROS in cancer cells^27^ likely due to their inability to fully oxidize hydroquinone. Complex III is highly conserved across phyla and of dual genetic origin^28^, with one of three main subunits (cytochrome *b*) mitochondrially encoded^29^, suggesting an evolutionary advantage in its conservation. Given the above, the safety profile of ATQ, the conservation of its binding pocket, and its demonstrated ability to induce ROS in cancer cells, we chose to repurpose ATQ as a candidate platinum agent sensitizer.

Using cisplatin and carboplatin, we report that ATQ induces chemosensitisation in vitro at pharmacologically achievable concentrations across a variety of cancer cell lines. Our mechanistic investigation revealed that ATQ induces mROS production, implicating ATQ-induced oxidative stress as mediator of the platinum sensitizing effect. To extend these findings, we show that the combination of ATQ with carboplatin delays the growth of avascular cancer cell spheroids. Taken together, our findings support further investigation of ATQ as a platinum sensitizing agent.

## Results

### Selection of ATQ concentration

ATQ has been shown in our previous work to inhibit OXPHOS in cancer cells by >90% at 30 μM ATQ within 2 h of treatment^30^. Previous studies have furthermore reported the induction of ROS in cancer cells at this physiologically achievable concentration^19–21,24^. We therefore selected 30 μM ATQ to use in the first instance for exploring potential platinum sensitization.

### ATQ Increases platinum-mediated cancer cell death in vitro

**Fig. 1:**
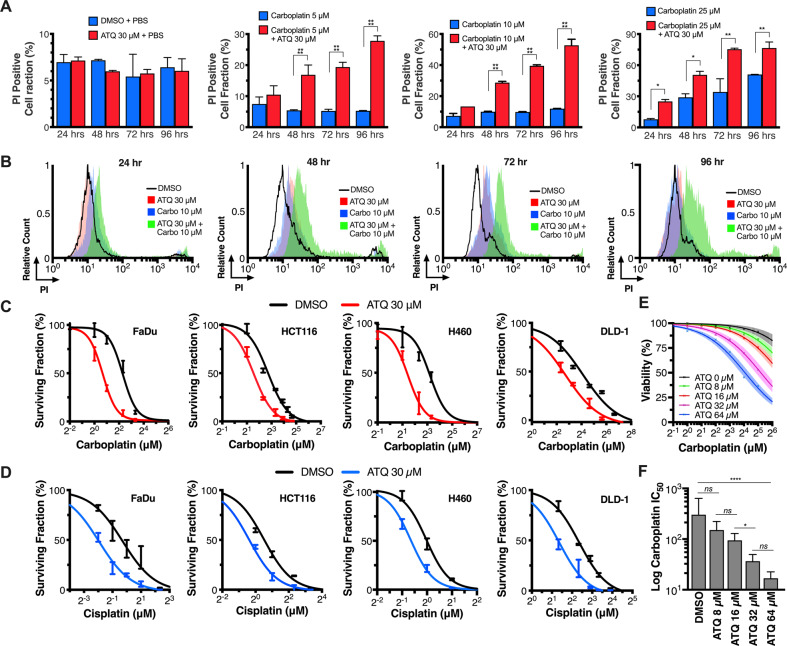
ATQ Potentiates Platinum-mediated Cancer Cell Death in vitro. **A** Propidium iodide (PI) exclusion as determined by flow cytometry. 1 × 10^5^ H460 cells were allowed to attach overnight, treated with DMSO or atovaquone (ATQ) for 2 h, then incubated concurrently with 0–25 μM carboplatin (as indicated) for an additional 24 h. Cells were then lifted and stained with ice-cold 50 μg/mL PI in PBS. **B** Representative histograms for (**A**). **C**, **D** Colony-forming assays (CFAs). Cells were plated and allowed to attach for 4 h, incubated 2 h with 30 μM ATQ or DMSO, then concurrently for 24 h with PBS or carboplatin. **E** Resazurin viability curves. H460 cells were plated and allowed to attach overnight, treated for 2 h with DMSO or ATQ, and then concurrently for 6 h with PBS or carboplatin. Resazurin was then added for 4 h. Fluorescence was measured at 550(ex)/590(em) nm. The viability of controls was set to 100%. A Loewe’s isobologram revealed supra-additivity (Fig. S2). **F** IC_50_ best-fit values from (**E**). Data representative of *N* = 3 experiments and shown with mean ± SD with significance determined using one- or two-way ANOVA according to: *P < 0.05; **P < 0.01; ***P < 0.001; ****P < 0.0001.

We first completed time-course PI-exclusion flow cytometry experiments for cell death in H460 (lung) cells using 30 μM ATQ and 0–25 μM carboplatin (Fig. 1A). We found minimal increases in PI-positive cell fraction over 24–96 h for DMSO controls and 30 μM ATQ treatments but observed strong concentration-dependent increases using ATQ in combination with carboplatin. This effect was repeated in FaDu (hypophareangeal) cells whereby a similar trend was found (Fig. S1A). Time-course histograms (Fig. 1B) expose the profound sensitizing effect that ATQ has in the presence of carboplatin. This striking, concentration- and time-dependent effect was recapitulated using cisplatin (Fig. S1B).

To expand on these findings, we completed colony formation assays (CFAs) with ATQ and carboplatin or cisplatin in additional cell lines (Fig. 1C, D). The combination resulted in potent chemosensitisation across all four cell lines with average relative IC_50_ values between DMSO and 30 µM ATQ (IC_50,Ratio_) of 0.357 for carboplatin and 0.499 for cisplatin (Table S1), i.e., 30 μM ATQ pre-treatment reduced the required concentration of carboplatin by 0.357^−1^ = 2.8-fold and 0.499^−1^ = 2.0-fold for cisplatin, on average.

To discern whether these effects may be specific to cancer cells, we completed CFAs with ATQ and carboplatin in normal tissue fibroblasts (Fig. S2), which revealed sensitization but to a lesser degree than in any of the cancer cell lines tested (*P* < 0.05). Importantly, all CFA experiments were performed in monolayer and under normoxia, suggesting a mechanism for chemosensitization that is independent of ATQ’s ability to alleviate hypoxia^30,31^.

To further probe the concentration-dependency of the sensitizing effect, we used a viability assay with a two-way dose-response for ATQ and carboplatin in H460 cells (Fig. 1E). The assay uses resazurin, a reliable redox-based metabolic indicator for viability with a positive midpoint potential which therefore does not perturb ETC dynamics, unlike tetrazolium reagents such as MTT^32^. Fitting a standard log-normal dose-response curve (Hill-slope = −1, we found a significant concentration reduction in carboplatin IC_50_ (IC_50,carbo_) across the range of concentrations tested (*P* < 0.0001; Fig. 1F), which was supra-additive (synergistic) according to Bliss independence (τ = 0.42–0.61). A Loewe’s isobologram also revealed synergy (combination index [CI] < 1) across the IC_50_ concentration ranges of ATQ and carboplatin (Fig. S3). We furthermore identified a plateau-threshold effect^33^ for the synergy having an inflection point between 16 and 32 μM ATQ (ATQ 8 μM vs. 16 μM: *P* > 0.05; ATQ 16 μM vs. 32 μM: *P* < 0.05; ATQ 32 μM vs. 64 μM: *P* > 0.05; Fig. 1E, F). In other words, log_2_-fold increases in ATQ concentrations outside of this range did not enhance sensitization. We therefore concluded that the underpinning mechanism for platinum sensitization by ATQ was triggered in a concentration-dependent manner across this threshold (16–32 μM ATQ).

### ATQ induces mROS production in cancer cells and is required for platinum sensitization

**Fig. 2:**
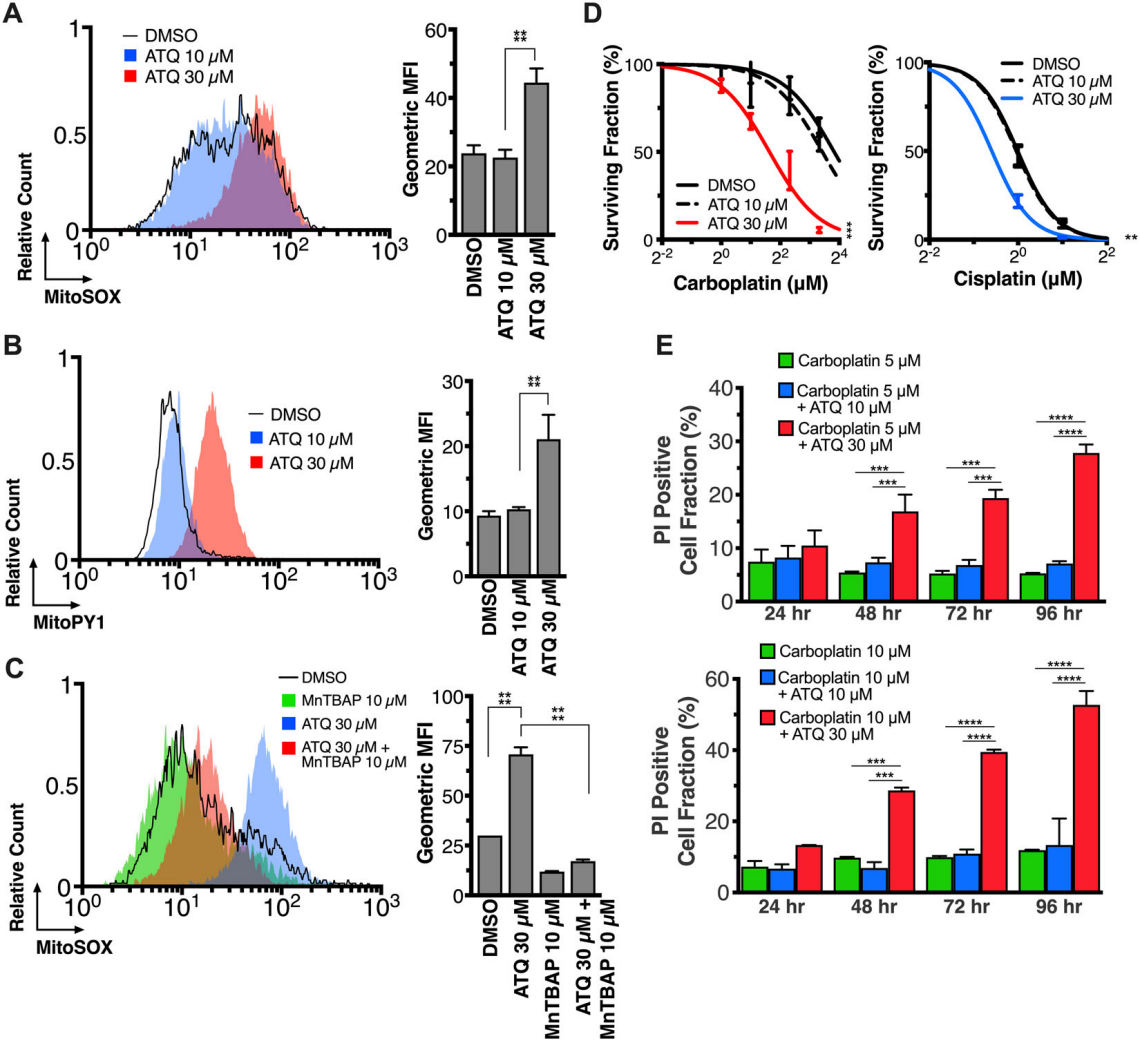
ATQ induces mROS and sensitizes cancer cells to platinums in a concentration-dependent manner. **A** Flow histograms of attached H460 cells treated for 30 min with MitoSOX after 2 h treatment with DMSO or ATQ. **B** Same as in A but using MitoPY1. **C** Cells were primed with MnTBAP at 10 μM for 2 h prior to treatment with DMSO or ATQ followed by flow for MitoSOX. **D** Colony-forming assays. H460 cells were allowed to attach and then treated for 2 h with butathione (BSO) followed by 24 h concomitant incubation with varying concentrations of platinums. Cells were then washed and allowed to form colonies for 14 days before crystal violet staining and quantification. **E** Time-course PI-exclusion using 0–30 μM ATQ or DMSO for 2 h followed by 24 h incubation with carboplatin 5 μM (top panel) or 10 μM (bottom). Cells were then lifted, stained with 50 μg/mL PI, and processed by flow at each timepoint. Data representative of at least *N* = 3 experiments and shown with mean ± SD. Significance determined using one- or two-way ANOVA according to: *P < 0.05; **P < 0.01; ***P < 0.001; ****P < 0.0001.

To probe whether the platinum sensitization by ATQ may be mediated by increased oxidative stress we used MitoSOX, a fluorescent indicator specific for mitochondrial superoxide (O_2_^−^.). We furthermore selected two concentrations of ATQ to work with for mechanistic investigations: a concentration beneath that required to induce sensitization (10 μM) and one at which we found potent sensitization (30 μM). We found no increase in superoxide production at 10 μM ATQ, but a striking increase in MitoSOX-positive cells was recorded at 30 μM ATQ in H460 (Fig. 2A) and FaDu (Fig. S4A) cells. We then evaluated ATQ-induced ROS generation using MitoPY1, a probe specific for hydrogen peroxide (a downstream product of SOD-mediated superoxide detoxification) that has a mitochondrial-targeting moiety^34^—we observed a similar increase in signal at 30 μM but not 10 μM ATQ (Figs. 2B and S4B). To further validate the increase in ROS, we obtained the cell-permeable mitochondrial superoxide dismutase 2 (SOD2) mimetic, MnTBAP, for use in rescue experiments. We selected 10 μM MnTBAP as a concentration that does not induce cytotoxicity but that has been shown to dismute H_2_O_2_ in human endothelial cells with high specificity^35^. Using MnTBAP, ATQ-induced mROS production was rescued in H460 (Fig. 2C) and FaDu (Fig. S5) cells. Taken together, these results suggest that ATQ induces mROS production in a concentration-dependent manner.

Next, we sought to confirm that the ability for ATQ to sensitize cancer cells to cisplatin or carboplatin was dependent on its ability to induce mROS (Fig. 2D). Indeed, we found that 30 μM ATQ, but not 10 μM ATQ, reduced survival of H460 cells treated with carboplatin or cisplatin. Similarly, time-course PI exclusion assays with carboplatin yielded no increase in PI-positive cell fraction at 10 μM ATQ but a clear increase at 30 μM ATQ (Fig. 2E). These findings demonstrate that a threshold concentration of ATQ is required to induce mROS production in order to sensitize cancer cells to cisplatin or carboplatin in vitro.

ATQ has been demonstrated to also be a potent inhibitor of STAT3^Y705^ phosphorylation^36^ and so we explored what role this may play in the context of platinum sensitization. Using DLD-1 cells isogenic for STAT3^Y705F/Y705F^, we found no observable differences in sensitivity to the combination of wild-type and mutant cells (Fig. S6A, B). We further investigated these results by combining platinum with STATTIC, an inhibitor of STAT3 dimerization, and found no sensitization in the absence of ATQ (Fig. S6C)^37^. Consequently, ATQ may be a potent and safe inhibitor of STAT3 but this does not mediate ATQ’s ability to induce platinum chemosensitisation in vitro.

### ATQ-mediated platinum sensitization is abrogated in vitro by preloading with the GSH Prodrug, NAC

**Fig. 3:**
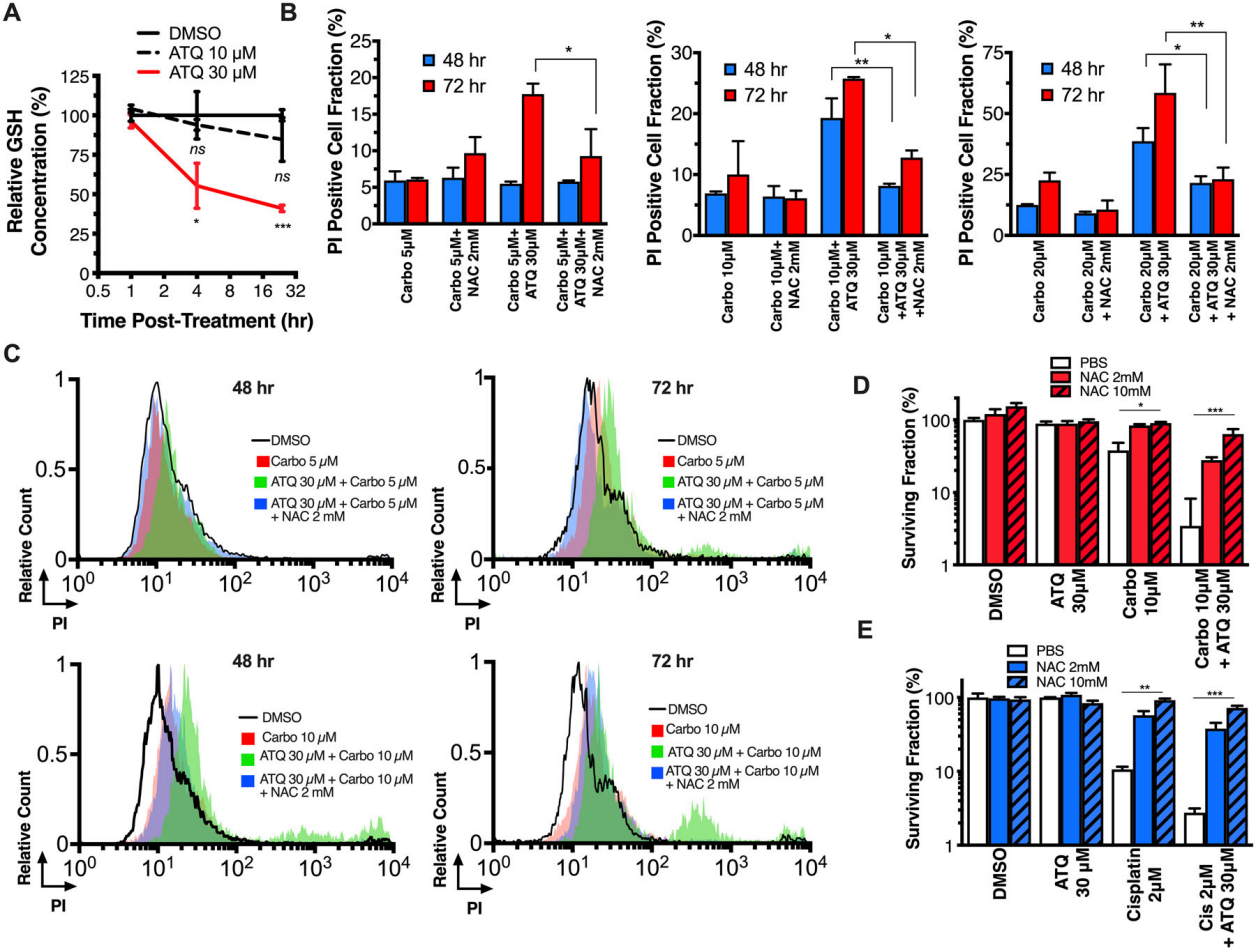
ATQ-mediated GSH pool depletion underpins platinum sensitization. **A** H460 cells were allowed to attach overnight then assayed for glutathione (GSH) using a colorimetric assay (Promega) at 1, 4, or 24 h post-ATQ treatment. **B** PI-exclusion time-course rescue flow assays. Attached H460 cells were preincubated for 2 h with 0–10 mM n-Acetyl cysteine (NAC), then 2 h co-incubation with 0–30 μM ATQ or DMSO, and then a further 24 h incubation with carboplatin 5 μM (left), 10 μM (middle) or 20 μM (right). **C** Representative histograms from (**B**). **D**, **E** Colony-forming assays using H460 cells. Cells were plated and allowed to attach for 4 h, pre-treated with 0–10 mM NAC for 2 h, then co-incubated for 2 h with 0–30 μM ATQ or DMSO, and then a further 24 h co-incubation with (**D**) carboplatin or (**E**) cisplatin. Data representative of *N* = 3 experiments and shown with mean ± SD. Significance determined using one-way ANOVA according to: *P < 0.05; **P < 0.01; ***P < 0.001; ****P < 0.0001.

Resistance to platinum-class agents has been shown to be mediated by intracellular levels of GSH, an antioxidant demonstrated to thwart platinum agent efficacy and toxicities in vitro and in vivo^3^. Conversely, GSH pool depletion has been shown to sensitize cancer cells to cisplatin and carboplatin^18^. We therefore hypothesized that ATQ-induced mROS production depletes GSH pools in a concentration-dependent manner and posited that this effect could underpin the sensitization. Accordingly, we measured intracellular GSH levels in H460 cells at 1, 4, and 24 h post-treatment using 10 or 30 μM ATQ (Fig. 3A). A significant decrease in GSH was observed using 30 μM ATQ but not 10 μM ATQ at 4 and at 24 h. We then used the specific inhibitor of GSH synthesis, buthionine sulfoximine (BSO), to validate that GSH pool depletion induces sensitization to cisplatin or carboplatin in our hands (Fig. S7). Indeed, the effect of both platinum agents was potentiated by BSO.

Having shown that ATQ treatment at 30 μM, but not 10 μM, induces mROS, depletes intracellular GSH pools, and sensitizes cancer cells to platinum-mediated cell death, we used the stable and well-characterized GSH precursor molecule, NAC, to investigate whether pre-loading cells prior to combination treatment could abrogate ATQ-mediated platinum sensitization. We found no potentiation of platinum-mediated cell death with 30 μM ATQ when cells were pre-loaded with 2- or 10-mM NAC (Fig. 3B, C and Fig. S8A). To confirm the abrogation of sensitization using NAC, CFAs were completed in H460 (Fig. 3D) and FaDu (Fig. S8B) cells. Indeed, the sensitizing effect was abrogated by NAC pre-treatment in a concentration-dependent manner and replicated using H460 cells and cisplatin (Fig. 3E). For further translational studies, carboplatin was selected as a representative platinum agent since it is better tolerated in vivo^38^.

### ATQ Potentiates carboplatin-induced growth delay of FaDu three-dimensional spheroids

We followed the growth of spheroids over time using brightfield microscopy to evaluate the effectiveness of cytotoxic or cytostatic treatments (a spheroid growth delay (SGD) assay). In contrast to xenograft experiments, SGDs can be done in a high-throughput manner to allow for broader dose-exploration.

**Fig. 4:**
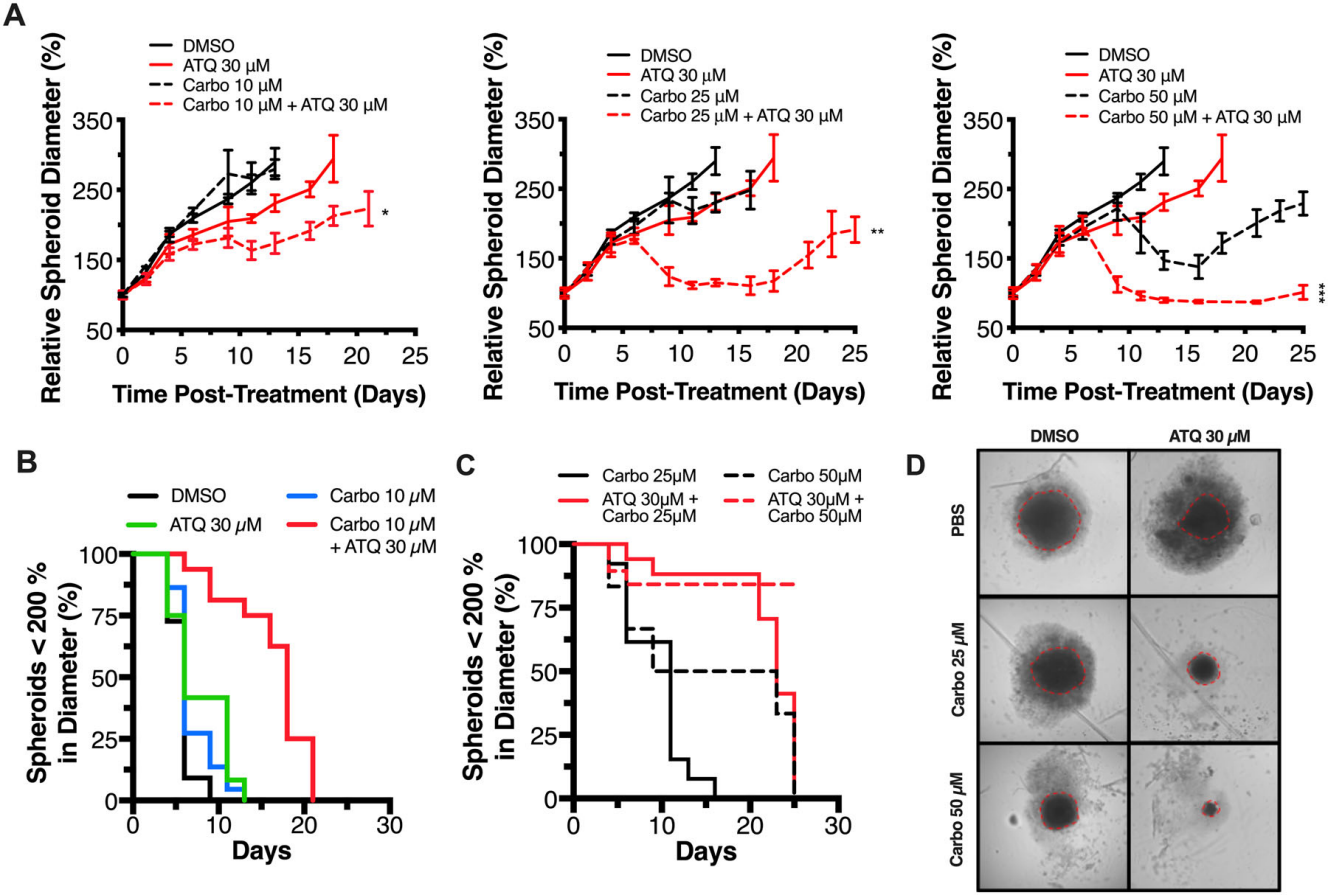
ATQ potentiates carboplatin-induced growth delay of 3D spheroids in a concentration-dependent manner. **A** Spheroid growth delay assay. FaDu cells (2 × 10^4^/well) were plated in ultra-low attachment 96-well plates (Corning) to form spheroids 550–650 μm in diameter and then treated for 24 h with ATQ or DMSO, then co-incubated for 24 h with PBS or carboplatin 10 μM (left), 25 μM (middle) or 50 μM (right). Spheroids were then washed, and growth monitored by brightfield microscopy. The medium was changed thrice weekly. Data points show the mean diameter relative to Day 0. Each condition used at least *N* = 16 spheroids. **B**, **C** Spheroid survival curves from experiments in (**A**) using a cutoff of ≥200 % of starting diameter as endpoint. **D** Day 17 microscopy of spheroids in (**A**) shown with optical density (OD) mask (red hashed lines). Data representative of *N* = 3 experiments and shown with mean ± SD. Significance determined using one-way ANOVA with significance defined according to: *P < 0.05; **P < 0.01; ***P < 0.001; ****P < 0.0001.

FaDu spheroids were generated and treated with DMSO or 30 µM ATQ and then 0–50 μM carboplatin before being imaged for up to 25 days (Fig. 4A). We found that ATQ potentiated the ability of carboplatin to delay spheroid growth as measured by optical density-guided spheroid diameter but had no significant effect as a single agent (Fig. 4B). Carboplatin at 10 µM significantly delayed spheroid growth (*P* < 0.01) (Fig. 4B) and this effect was increased at higher concentrations of carboplatin (Fig. 4C). Indeed, high dose carboplatin at 50 µM inhibited spheroid growth as a single agent though this may be less physiologically relevant. Representative microscopy images of spheroids from Fig. 4A–C on Day 17 can be seen in Fig. 4D.

## Discussion

We demonstrated that ATQ potentiates the anti-cancer effects of cisplatin and carboplatin in two-dimensional CFAs and PI-exclusion assays, an effect mediated by increased oxidative stress due to mROS induction confirmed by concurrent GSH pool depletion. We rescued the increase in ROS using MnTBAP, which mimics mitochondrial superoxide dismutase, and used the GSH precursor NAC to abrogate the sensitizing effect through the restoration of GSH pools. Together, our findings strongly implicate increased oxidative stress (mROS) as the key effect mediator. ATQ and carboplatin also synergistically inhibited the growth of spheroids in a concentration-dependent manner. ATQ is also concurrently being repurposed as a modifier of radiobiological tumor hypoxia (Atovaquone as Tumor HypOxia Modifier (ATOM) trial; NCT02628080) by our team. As such, and because radiotherapy is markedly enhanced by adequate tumor oxygenation, the combined effect of alleviating hypoxia and sensitizing to platinum agents could provide opportunities for ATQ to be used clinically in any number of scenarios. Accordingly, our team is now also evaluating ATQ in combination with radiation and platinum-based chemotherapy in a multi-center Phase I setting for the treatment of NSLSC (trial in set up).

We observed repeatable and striking concentration- and time-dependent sensitization in vitro across a variety of cancer cell lines, however, the degree of sensitization was variable between cell lines (Table S1). The differences in sensitivity between cell lines may reflect differences in lineage and functional mutations, possibly leading to differences in basal GSH or mROS, which were not investigated in the present work. Notably, we found that DLD-1 cells were relatively resistant to platinums as single agents and H460 cells relatively sensitive to carboplatin in the presence of ATQ.

As a molecule, ATQ is highly lipophilic, leading to heavy protein binding (>99.9%) in the bloodstream, a prolonged circulatory half-life, and is practically insoluble in aqueous solutions^39,40^. Its poor aqueous solubility may be improved using alternative formulations such as liposomal encapsulations^41^, which, coupled with ATQ’s favorable tolerability as an anti-malarial, may also facilitate higher steady-state concentrations. At the same time, a meta-analysis of ATQ bioavailability found that inter-patient variability across six malaria trials was 107%^23^. Therefore, analyses of individual patient steady-state plasma levels may be required for optimal translation of ATQ into the clinic, albeit as a platinum sensitizer or hypoxia modifier.

Platinums are effective anti-cancer agents but known to provide narrow therapeutic windows due to dose-limiting hematopoietic toxicities. Strategies to improve their tolerability could therefore be beneficial for platinum-based combination therapies. Reformulations include liposomal encapsulation^42,43^, which protract circulating half-life and allow modifications to target tumors more precisely. Similarly, a reformulation of ATQ through the use of nanoparticles has been shown to provide mice adequate protection against malaria for up to a month without readministration^44^ and could be explored for use with platinum chemotherapy. Approaches to reduce normal tissue toxicity or optimize pharmacokinetics may provide advantages for single agents but remain to be evaluated in a combination setting.

Under homeostatic conditions, cells control ROS levels by balancing ROS-inducing metabolic activities with its elimination. Under increased oxidative stress, however, excessive ROS can catastrophically damage cells. In this context, cancer cells have been shown to exhibit a greater level of basal oxidative stress^45^. Preclinical studies have demonstrated that cancer cells are more sensitive to critical levels of oxidative stress compared to normal cells, which may provide an opportunity for a therapeutic window^46^. Oxidative stress has also been heavily implicated in mediating platinum agent toxicities^47^ and so subsequent studies evaluating the translational relevance of OXPHOS inhibitors should consider normal tissue effects.

Downstream effects of agents that modulate oxidative stress are complex and diverse. Incomplete knowledge of the role that oxidative stress plays in promoting or limiting disease progression has likely limited the success of OXPHOS-based treatments to-date. To help resolve this, future clinical studies could evaluate the evolution of cellular metabolism in patients during platinum treatment. This may shed light on whether enduring, resistant, clonogenically capable cancer cells have more reliance on OXPHOS or increased sensitivity to ROS in situ. If validated, such findings would provide significant, clinically actionable evidence for combining OXPHOS inhibitors like ATQ with platinum agents by way of a therapeutic index.

In light of the cost-effectiveness of platinum agents, their ubiquity in the clinic, the conservation of ATQ’s mitochondrial binding pocket, and the tolerability of ATQ as an anti-malarial drug, our findings suggest that the combination of ATQ and platinum agents is a translationally relevant treatment strategy worthy of further investigation.

## Materials and methods

### Tissue culture

HCT116 (colorectal), FaDu (hypopharengeal), H460 (lung), and DLD-1 (colorectal) cell lines were purchased from American Type Culture Collection (ATCC). HCT116, FaDu, and DLD-1 cells were maintained in Dulbecco’s modified Eagle’s medium (DMEM, Sigma) high-glucose supplemented with 10% fetal bovine serum (FBS) and 100 U penicillin + 100 µg/mL streptomycin. H460 cell lines were maintained in RPMI-1640 media (Sigma) supplemented with 10% FBS and 100 U penicillin + 100 µg/mL streptomycin. All cell lines were authenticated by LGC standards (ATCC) by short tandem repeat (STR) profiling and tested for mycoplasma using MycoAlert (Lonza). Cell lines were maintained in culture at 37 °C supplied with 5% CO_2_.

### Drug treatment

Cisplatin (1134357, Sigma) and carboplatin (C2538, Sigma) were reconstituted immediately prior to each use in 0.9% NaCl_aq_ since DMSO readily inactivates platinum agents^48^. Platinum treatments were performed in all experiments by adding 20× concentrated solution in medium to each well for 24 h. ATQ (A7986, Sigma) was constituted to 100 mM in DMSO for stock solutions and stored at −80 °C. For working, ATQ was then diluted in PBS yielding <0.3% DMSO. For combination treatment, cells were pre-incubated with ATQ for 2 h followed by incubation with platinum in NaCl_aq_ for 24 h. Combination rescue experiments pre-loaded of cells with fresh NAC (Sigma) in PBS for 2 h followed by ATQ then platinum, as above.

### Colony formation assays

Cells were lifted in trypsin, counted and re-plated as single cells in 6-well plates. Cells were allowed to attach for 4 h and then treated as indicated for 24 h. After compound removal, colonies were grown for ~14 days, fixed and stained with crystal violet in methanol (MeOH), and then counted using a GelCount colony counter (Oxford Optronix). Plated cell numbers for each experiment are described within their respective figures.

### Propidium iodide viability stain

Cells were harvested by trypsinization and centrifugation, including those that were non-adherent at that time. Cell pellets were washed with 1 mL of ice-cold 1× PBS and resuspended in 50 μg/mL propidium iodide (PI) (Sigma) in PBS. Samples were kept on ice and away from light, gently vortexed, and then immediately analyzed via flow cytometry.

### Viability assay

Viability was assayed using resazurin (R7017, Sigma) and performed in white-walled 96-well microplates using a standard protocol^32^. H460 cells were seeded at a density of 0.5 × 10^4^ per well and allowed to attach for four hours and protected from light, then treated with DMSO vehicle or ATQ for 2 h. Following this, cells were co-treated with PBS (control) or carboplatin for 4 h. Resazurin was then added for 4 h before fluorescence intensity was measured at excitation/emission wavelengths of 550/590 nm. The viability of controls was set to 100%.

### Live fluorescent mitochondrial ROS indicators

MitoSOX (ab219943, Abcam) and MitoPY1 (SML0734, Sigma) were stored at −80 °C and dissolved in DMSO to stock concentrations of 5 mM. Cells were counted and plated in 6-well plates at 0.5 × 10^4^ cells per well and then allowed to attach for 4 h at 37 °C prior to being incubated with 10 μM MitoPY1 or 5 μM MitoSOX for 30 min. DMSO or 30 μM ATQ was then added at 20× concentration and cells were incubated for a further 2 h. Cells were then harvested by trypsinization, centrifuged at 200 × *g* for 5 min, and resuspended in 200-μL ice-cold PBS. Samples were run on a FACSCalibur (BD Biosciences). Fluorescence was quantified by geometric mean.

### Three-dimensional tissue culture (Spheroids)

FaDu cells were plated in ultra-low attachment U-bottom plates (Corning, #7007) at a density of 1 × 10^4^ cells per well in low-glucose DMEM medium (Sigma) and supplemented with 10% FBS and 200 μM pyruvate (Sigma). Three-dimensional spheroid formation was inspected manually and confirmed using microscopy. After 72 h, the spheroid diameter was recorded daily and derived from cross-sectional mid-plane measurements acquired with a GelCount Colony Counter (Oxford Optronix). Once spheroids reached 550–650 μm in diameter, they were treated for 24 h as indicated in each experiment with at least *N* = 16 spheroids per condition.

### SGD assay

FaDu cells were seeded to generate spheroids, as described above. Once spheroids reached 550–650 μm in diameter, half the medium of each well (100 μL) was removed and replaced with 2× ATQ 30 μM or concentration volume-matched control solvent (DMSO). Each plate contained a DMSO control and each ATQ-treated plate contained DMSO and ATQ controls to account for plate-to-plate variations. Each treatment group contained at least 16 separate spheroids. For drugging, attention was paid to limit perturbations to the spheroids while in culture. Plates were then incubated for a further 24 h. Drugs were then washed off via serial medium change (3×). Spheroids were observed and diameter measurements were recorded thrice a week using a high-throughput amenable 96-well plate brightfield microscope (Celligo, Nexcelcom). Half the volume of the medium of each spheroid well was changed thrice weekly.

### Analysis and quantification

Significance of differences between means was measured by *t*-test or one-way ANOVA with Bonferroni or Tukey post-tests (when applicable) using Prism 8.0 (GraphPad, USA). Measurements were expressed as mean ± SD. Significance was determined according to two-tailed *P*-values. Chemosensitization to platinum agents was quantified by taking the ratio of the IC_50_ for DMSO control to ATQ-treated groups (IC_50,ratio_). In the case of resazurin-based viability assay, Bliss independence, Loewe’s, or Chou-Talalay were used to quantify synergy^49–51^.

## Supplementary information

Supplementary Figure Legends & Table Captions

Figure S1. ATQ Potentiates Platinum-mediated Cancer Cell Death in vitro

Figure S2. ATQ Sensitizes Normal Fibroblasts in vitro to Carboplatin

Figure S3. Normalized Loewe’s Isobologram of ATQ in Combination with Carboplatin in vitro

Figure S4. ATQ Induces mROS in FaDu Cells

Figure S5. MnTBAP Partially Abrogates ATQ-induced mROS in FaDu Cells

Figure S6. Inhibition of pSTAT3Y705F does not Mediate ATQ-induced Platinum Sensitization

Figure S7. BSO Sensitizes H460 Cells to Platinums in vitro

Figure S8. NAC Rescues ATQ-induced Platinum Sensitization in FaDu Cells in vitro

Table S1. Atovaquone-induced IC50 Shifts in Clonogenic Survival for Platinum Agents

## References

[1] Kelland, L. The resurgence of platinum-based cancer chemotherapy. *Nat. Rev. Cancer.*10.1038/nrc2167 (2007). 10.1038/nrc2167 17625587

[2] Makovec, T. Cisplatin and beyond: in cancer chemotherapy. *Radiol. Oncol.*10.2478/raon-2019-0018 (2019). 10.2478/raon-2019-0018 PMC6572495 30956230

[3] Galluzzi L (2012). Molecular mechanisms of cisplatin resistance. Oncogene.

[4] D’Addario, G. et al. Platinum-based versus non-platinum-based chemotherapy in advanced non-small-cell lung cancer: a meta-analysis of the published literature. *J. Clin. Oncol.*10.1200/JCO.2005.03.045 (2005). 10.1200/JCO.2005.03.045 15728229

[5] Basourakos, S. P. et al. Combination platinum-based and DNA damage response-targeting cancer therapy: evolution and future directions. *Curr. Med. Chem.*10.2174/0929867323666161214114948 (2016). 10.2174/0929867323666161214114948 PMC5471128 27978798

[6] Rugo, H. S. et al. Adaptive randomization of veliparib-carboplatin treatment in breast cancer. *N. Engl. J. Med.*10.1056/NEJMoa1513749 (2016). 10.1056/NEJMoa1513749 PMC5259561 27406347

[7] Pfisterer, J. et al. Bevacizumab and platinum-based combinations for recurrent ovarian cancer: a randomised, open-label, phase 3 trial. *Lancet Oncol.*10.1016/S1470-2045(20)30142-X (2020). 10.1016/S1470-2045(20)30142-X 32305099

[8] Fennell, D. A. et al. Cisplatin in the modern era: the backbone of first-line chemotherapy for non-small cell lung cancer. *Cancer Treat. Rev.*10.1016/j.ctrv.2016.01.003 (2016). 10.1016/j.ctrv.2016.01.003 26866673

[9] Dilruba, S. & Kalayda, G. V. Platinum-based drugs: past, present and future. *Cancer Chemother. Pharmacol.*10.1007/s00280-016-2976-z (2016). 10.1007/s00280-016-2976-z 26886018

[10] Rosenberg, B., VanCamp, L., Trosko, J. E. & Mansour, V. H. Platinum compounds: a new class of potent antitumour agents. *Nature*. 10.1038/222385a0 (1969). 10.1038/222385a0 5782119

[11] Wang, D. & Lippard, S. J. Cellular processing of platinum anticancer drugs. *Nat. Rev. Drug Discov.*10.1038/nrd1691 (2005). 10.1038/nrd1691 15789122

[12] Siddik ZH (2003). Cisplatin: mode of cytotoxic action and molecular basis of resistance. Oncogene.

[13] Atsushi H, Shuji S, Kosuke A, Takafumi K (1994). A comparison of in vitro platinum-DNA adduct formation between carboplatin and cisplatin. Int. J. Biochem..

[14] Bruno PM (2017). A subset of platinum-containing chemotherapeutic agents kills cells by inducing ribosome biogenesis stress. Nat. Med..

[15] Inapurapu S, Kudle KR, Bodiga S, Bodiga VL (2017). Cisplatin cytotoxicity is dependent on mitochondrial respiration in *Saccharomyces cerevisiae*. Iran J. Basic Med. Sci..

[16] He, P. J. et al. Oxidative stress induced by carboplatin promotes apoptosis and inhibits migration of HN-3 cells. *Oncol. Lett*. 10.3892/ol.2018.9563 (2018). 10.3892/ol.2018.9563 PMC6256460 30546448

[17] Marullo R (2013). Cisplatin induces a mitochondrial-ros response that contributes to cytotoxicity depending on mitochondrial redox status and bioenergetic functions. PLoS ONE.

[18] Sluiter WJ, Mulder NH, Timmer-Bosscha H, Jan Meersma G, de Vries EGE (1992). Relationship of cellular glutathione to the cytotoxicity and resistance of seven platinum compounds. Cancer Res..

[19] Das S, Dielschneider R, Chanas-LaRue A, Johnston JB, Gibson SB (2018). Antimalarial drugs trigger lysosome-mediated cell death in chronic lymphocytic leukemia (CLL) cells. Leuk. Res..

[20] Druck, T. et al. Fhit–Fdxr interaction in the mitochondria: modulation of reactive oxygen species generation and apoptosis in cancer cells. *Cell Death Dis*. 10.1038/s41419-019-1414-7 (2019). 10.1038/s41419-019-1414-7 PMC6377664 30770797

[21] Ke F (2018). The anti-malarial atovaquone selectively increases chemosensitivity in retinoblastoma via mitochondrial dysfunction-dependent oxidative damage and Akt/AMPK/mTOR inhibition. Biochem. Biophys. Res. Commun..

[22] Sun, Y., Xu, H., Chen, X., Li, X. & Luo, B. Inhibition of mitochondrial respiration overcomes hepatocellular carcinoma chemoresistance. *Biochem. Biophys. Res. Commun*. 10.1016/j.bbrc.2018.11.182 (2019). 10.1016/j.bbrc.2018.11.182 30522865

[23] Nixon GL (2013). Antimalarial pharmacology and therapeutics of atovaquone. J. Antimicrob. Chemother..

[24] Fiorillo M (2016). Repurposing atovaquone: targeting mitochondrial complex III and OXPHOS to eradicate cancer stem cells. Oncotarget.

[25] Capper, M. J. et al. Antimalarial 4(1H)-pyridones bind to the Qi site of cytochrome bc1. *Proc. Natl Acad. Sci. USA*. 10.1073/pnas.1416611112 (2015). 10.1073/pnas.1416611112 PMC4311836 25564664

[26] Birth D, Kao W-C, Hunte C (2014). Structural analysis of atovaquone-inhibited cytochrome bc1 complex reveals the molecular basis of antimalarial drug action. Nat. Commun..

[27] Lee, D. W. et al. Loss of a conserved tyrosine residue of cytochrome b induces reactive oxygen species production by cytochrome bc1. *J. Biol. Chem.*10.1074/jbc.M110.214460 (2011). 10.1074/jbc.M110.214460 PMC3093886 21454570

[28] Smith, P. M., Fox, J. L. & Winge, D. R. Biogenesis of the cytochrome bc 1 complex and role of assembly factors. *Biochimica et Biophysica Acta - Bioenergetics.*10.1016/j.bbabio.2011.11.009 (2012). 10.1016/j.bbabio.2011.11.009 PMC3366459 22138626

[29] Howell, N. Evolutionary conservation of protein regions in the protonmotive cytochrome b and their possible roles in redox catalysis. *J. Mol. Evol*. 10.1007/BF02100114 (1989). 10.1007/BF02100114 2509716

[30] Ashton TM (2016). The anti-malarial atovaquone increases radiosensitivity by alleviating tumour hypoxia. Nat. Commun..

[31] Ashton, T. M. et al. Oxidative phosphorylation as an emerging target in cancer therapy. *Clin. Cancer Res*. 10.1158/1078-0432.CCR-17-3070 (2018). 10.1158/1078-0432.CCR-17-3070 29420223

[32] O’Brien, J., Wilson, I., Orton, T. & Pognan, F. Investigation of the Alamar Blue (resazurin) fluorescent dye for the assessment of mammalian cell cytotoxicity. *Eur. J. Biochem*. 10.1046/j.1432-1327.2000.01606.x (2000). 10.1046/j.1432-1327.2000.01606.x 10951200

[33] Ivanova, A. & Xiao, C. Dose finding when the target dose is on a plateau of a dose-response curve: comparison of fully sequential designs. *Pharm. Stat*. 10.1002/pst.1585 (2013). 10.1002/pst.1585 PMC3770738 23893900

[34] Dickinson, B. C. & Chang, C. J. A targetable fluorescent probe for imaging hydrogen peroxide in the mitochondria of living cells. *J. Am. Chem. Soc*. 10.1021/ja802355u (2008). 10.1021/ja802355u PMC2810491 18605728

[35] Day, B. J., Fridovich, I. & Crapo, J. D. Manganic porphyrins possess catalase activity and protect endothelial cells against hydrogen peroxide-mediated injury. *Arch. Biochem. Biophys*. 10.1006/abbi.1997.0341 (1997). 10.1006/abbi.1997.0341 9367533

[36] Xiang M (2016). Gene expression-based discovery of atovaquone as a STAT3 inhibitor and anti-cancer agent. Blood.

[37] Schust, J., Sperl, B., Hollis, A., Mayer, T. U. & Berg, T. Stattic: a small-molecule inhibitor of STAT3 activation and dimerization. *Chem. Biol*. 10.1016/j.chembiol.2006.09.018 (2006). 10.1016/j.chembiol.2006.09.018 17114005

[38] Boven E, Nauta MM, Schluper HMM, Pinedo HM, van der Vijgh WJF (1985). Comparative activity and distribution studies of five platinum analogues in nude mice bearing human ovarian carcinoma xenografts. Cancer Res..

[39] Gary, R. Greenstein The Merck Index, An Enclyopedia of Chemicals, Drugs, and Biologicals (14th Ed.). *The Merck Index* (2007).

[40] Zsila, F. & Fitos, I. Combination of chiroptical, absorption and fluorescence spectroscopic methods reveals multiple, hydrophobicity-driven human serum albumin binding of the antimalarial atovaquone and related hydroxynaphthoquinone compounds. *Org. Biomol. Chem*. 10.1039/c0ob00124d (2010). 10.1039/c0ob00124d 20737064

[41] Cauchetier, E. et al. Therapeutic evaluation of free and liposome-encapsulated atovaquone in the treatment of murine leishmaniasis. *Int. J. Parasitol*. 10.1016/S0020-7519(00)00053-9 (2000). 10.1016/s0020-7519(00)00053-9 10856513

[42] Liu, D., He, C., Wang, A. Z. & Lin, W. Application of liposomal technologies for delivery of platinum analogs in oncology. *Int. J. Nanomed.*10.2147/IJN.S38354 (2013). 10.2147/IJN.S38354 PMC3767488 24023517

[43] Catanzaro, D. et al. Cisplatin liposome and 6-amino nicotinamide combination to overcome drug resistance in ovarian cancer cells. *Oncotarget.*10.18632/oncotarget.24708 (2018). 10.18632/oncotarget.24708 PMC5908290 29682189

[44] Bakshi, R. P. et al. Long-acting injectable atovaquone nanomedicines for malaria prophylaxis. *Nat. Commun*. 10.1038/s41467-017-02603-z (2018). 10.1038/s41467-017-02603-z PMC5778127 29358624

[45] Schumacker, P. T. Reactive oxygen species in cancer cells: Live by the sword, die by the sword. *Cancer Cell*. 10.1016/j.ccr.2006.08.015 (2006). 10.1016/j.ccr.2006.08.015 16959608

[46] Raj, L. et al. Selective killing of cancer cells by a small molecule targeting the stress response to ROS. *Nature*10.1038/nature10167 (2011). 10.1038/nature10167 PMC3316487 21753854

[47] Martins, N. M., Santos, N. A. G., Curti, C., Bianchi, M. L. P. & Dos Santos, A. C. Cisplatin induces mitochondrial oxidative stress with resultant energetic metabolism impairment, membrane rigidification and apoptosis in rat liver. *J. Appl. Toxicol*. 10.1002/jat.1284 (2008). 10.1002/jat.1284 17604343

[48] Hall, M. D. et al. Say no to DMSO: Dimethylsulfoxide inactivates cisplatin, carboplatin, and other platinum complexes. *Cancer Res*. 10.1158/0008-5472.CAN-14-0247 (2014). 10.1158/0008-5472.CAN-14-0247 PMC4153432 24812268

[49] Zhao, W. et al. A new bliss independence model to analyze drug combination data. *J. Biomol. Screen*. 10.1177/1087057114521867 (2014). 10.1177/1087057114521867 24492921

[50] Liu, Q., Yin, X., Languino, L. R. & Altieri, D. C. Evaluation of drug combination effect using a bliss independence dose–response surface model. *Stat. Biopharm. Res*. 10.1080/19466315.2018.1437071 (2018). 10.1080/19466315.2018.1437071 PMC6415926 30881603

[51] Chou, T. C. Drug combination studies and their synergy quantification using the chou-talalay method. *Cancer Res.*10.1158/0008-5472.CAN-09-1947 (2010). 10.1158/0008-5472.CAN-09-1947 20068163

