# Supplementary material for: The anti-malarial drug atovaquone potentiates platinum-mediated cancer cell death by increasing oxidative stress
Source: Cell Death Discov. 2020 Oct 27;6:110. doi: 10.1038/s41420-020-00343-6 (PMC7591508; doi:10.1038/s41420-020-00343-6)
Supplement: Supplementary file 1 — Supplementary Figure Legends & Table Captions [file 41420_2020_343_MOESM1_ESM.docx]

# Supplementary Legends & Captions

**Figure S1. ATQ Potentiates Platinum-mediated Cancer Cell Death *in vitro***

**(A,B)** Propidium iodide (PI) exclusion flow cytometry for quantifying cell death. (A) 1x10^5^ FaDu or (B) H460 cells were plated in triplicate and allowed to attach overnight, treated with 30 μM Atovaquone (ATQ) for 2 hr, then incubated concurrently with 0-25 μM carboplatin (A) or 0-2 μM cisplatin (B) for an additional 24 hr. Medium was then replaced and cells were incubated for 24-96 hr and then lifted and stained with ice cold 50 μg/mL PI in PBS at each respective timepoint. Data representative of at least *N* = 3 experiments and shown with mean ± SD with significance determined using one-way ANOVA according to: **P*<0.05; ***P*<0.01; ****P*<0.001; *****P*<0.0001.

**Table S1. Atovaquone-induced IC_50_ Shifts in Clonogenic Survival for Platinum Agents.**

**Figure S2. ATQ Sensitizes Normal Fibroblasts *in vitro* to Carboplatin**

Colony forming assays (CFA) performed in triplicate in MRC5. Cells were plated and allowed to attach for 4 hr and then pretreated with DMSO or ATQ 30 μM for 2 hr then co-incubated with carboplatin for a further 24 hrs. Data representative of *N*=3. Errors bars are SD.

**Figure S3. Normalized Loewe’s Isobologram of ATQ in Combination with Carboplatin *in vitro***

H460 cells were plated and allowed to attach overnight, treated for 2 hr with DMSO or ATQ, and then concurrently for 6 hr with PBS or carboplatin. Resazurin was then added for 4 hr. Fluorescence was measured at 550(ex)/590(em) nm. CI = combination index.

**Figure S4. ATQ Induces mROS in FaDu Cells**

**(A)** Flow histograms of attached FaDu cells left to attach overnight then treated for 30 min with MitoSOX after 2 hr treatment with DMSO or ATQ. **(B)** Same as in A but using MitoPY1.

**Figure S5. MnTBAP Partially Abrogates ATQ-induced mROS in FaDu Cells**

FaDu cells were plated and allowed to attach overnight. Cells were then primed with the MnSOD mimetic MnTBAP at 10 μM for 2 hr prior to treatment with DMSO or ATQ followed by flow for MitoSOX.

**Figure S6. Inhibition of pSTAT3^Y705F^ does not Mediate ATQ-induced Platinum Sensitization.**

**(A,B)** Colony forming assays (CFA) performed in triplicate using DLD-1 (colorectal) cells isogenic for Y705F, which abrogates ATQ-mediated phospho-inhibition. Cells were plated and allowed to attach for 4 hr and then pretreated with DMSO or ATQ 30 μM for 2 hr and then co-incubated with carboplatin (A) or cisplatin (B). Sensitization was recorded in the case of both carboplatin/cisplatin for wild-type cells (*P*<0.0001) and STAT3 mutant cells (*P*<0.001). **(C)** To further exclude any role for STAT3, we used STATTIC to inhibit prevent STAT3 dimerization and then performed CFAs as in (A,B) and identified no significant differences in sensitization across the dose-range tested.

**Figure S7. BSO Sensitizes H460 Cells to Platinums *in vitro***

H460 cells were plated and allowed to attach overnight. Cells were then primed with the potent and specific GSH inhibitor buthionine sulphoximine (BSO) for 2 hr prior to treatment with DMSO or ATQ for a further 2 hr. Carboplatin (left) or cisplatin (right) was then added for 24 hr. Media was replaced and colonies were allowed to form for 14 days. To quantify colonies, methanol-suspended crystal violet staining was then performed. Best-fit IC_50_ shifts for carboplatin (*P*<0.0001) and cisplatin (*P*<0.001) were 0.397 and 0.518, respectively.

**Figure S8. NAC Rescues ATQ-induced Platinum Sensitization in FaDu Cells *in vitro***

**(A)** FaDu cells were plated and allowed to attach overnight. Cells were then primed with the GSH pro-drug N-acetyl cysteine (NAC) for 2 hr prior to treatment with DMSO or ATQ for a further 2 hr. Carboplatin or PBS was then added for a further 24 hr. Media was then replaced. Cells were lifted, stained with 50 μg/mL PI, and processed by flow at each timepoint. **(B)** FaDu cells were plated and allowed to attach for 4 hr before priming with NAC or PBS, following by treatment for 2 hr with DMSO or 30 μM ATQ. PBS vehicle or carboplatin was then added to each well and cells were further incubated for 24 hr. Media was then replaced and colonies were allowed to form for 14 days. To quantify colonies, methanol-suspended crystal violet staining was then performed. Data representative of at least *N*=3 repeats and is mean +/- sd.
