# Supplementary figures and images for: The anti-malarial drug atovaquone potentiates platinum-mediated cancer cell death by increasing oxidative stress
Source: Cell Death Discov. 2020 Oct 27;6:110. doi: 10.1038/s41420-020-00343-6 (PMC7591508; doi:10.1038/s41420-020-00343-6)

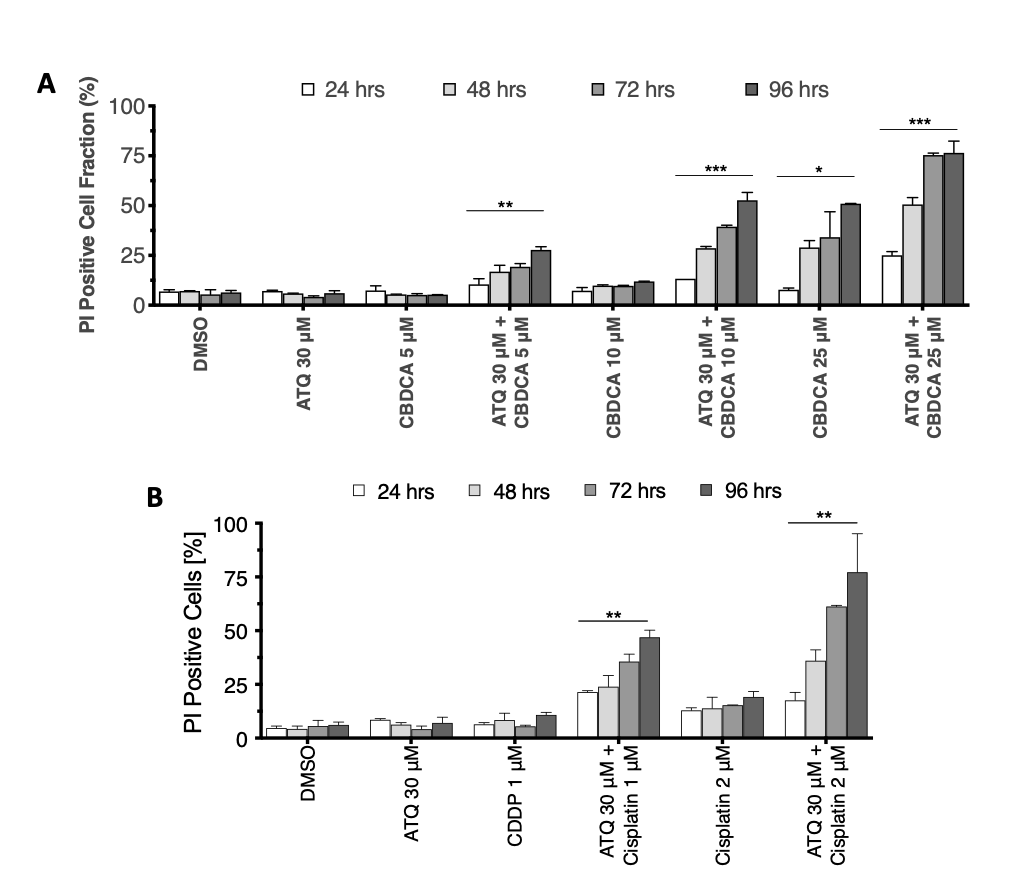

Supplement: Supplementary file 2 — Figure S1. ATQ Potentiates Platinum-mediated Cancer Cell Death in vitro [file 41420_2020_343_MOESM2_ESM.png]

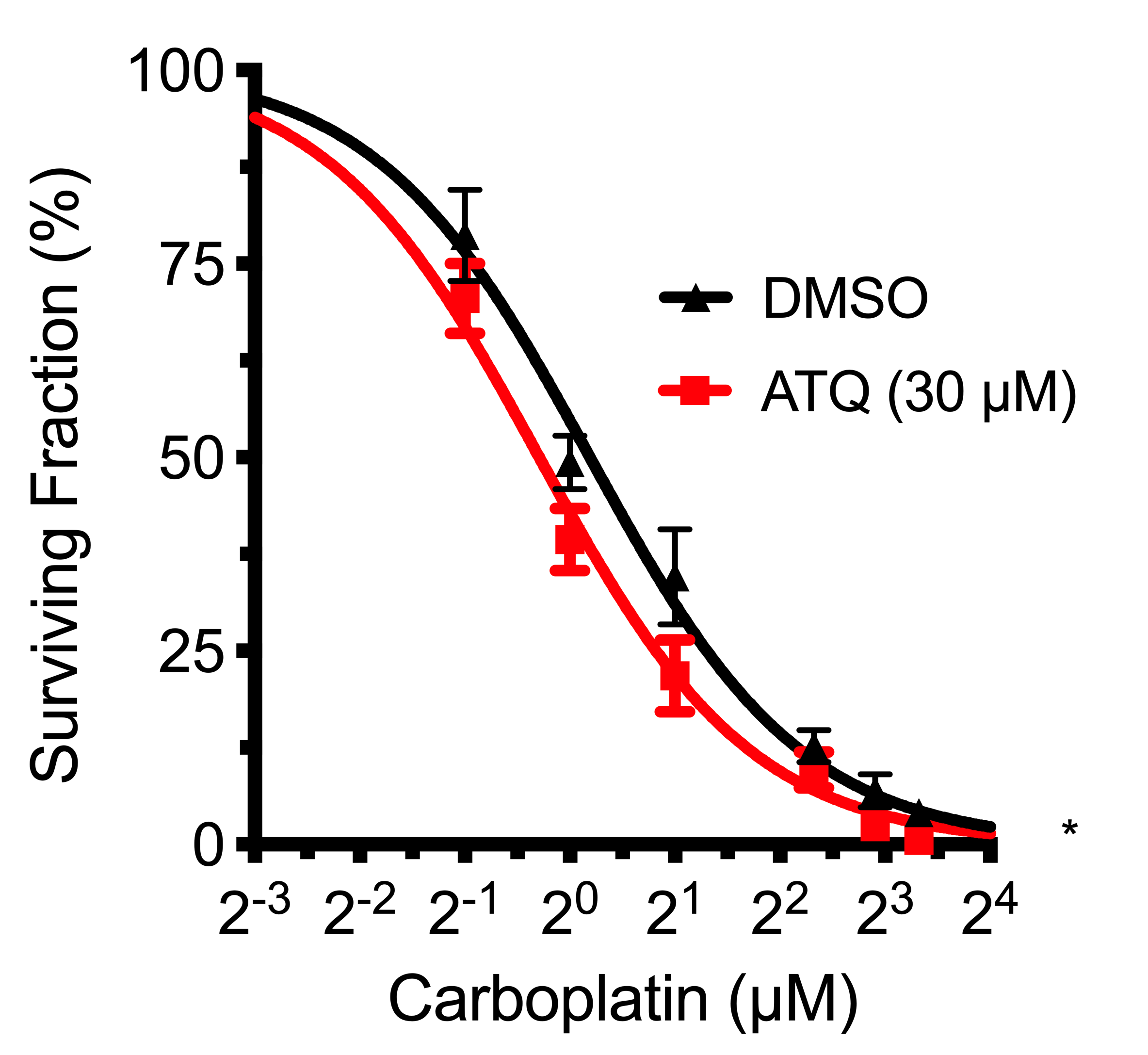

Supplement: Supplementary file 3 — Figure S2. ATQ Sensitizes Normal Fibroblasts in vitro to Carboplatin [file 41420_2020_343_MOESM3_ESM.tif]

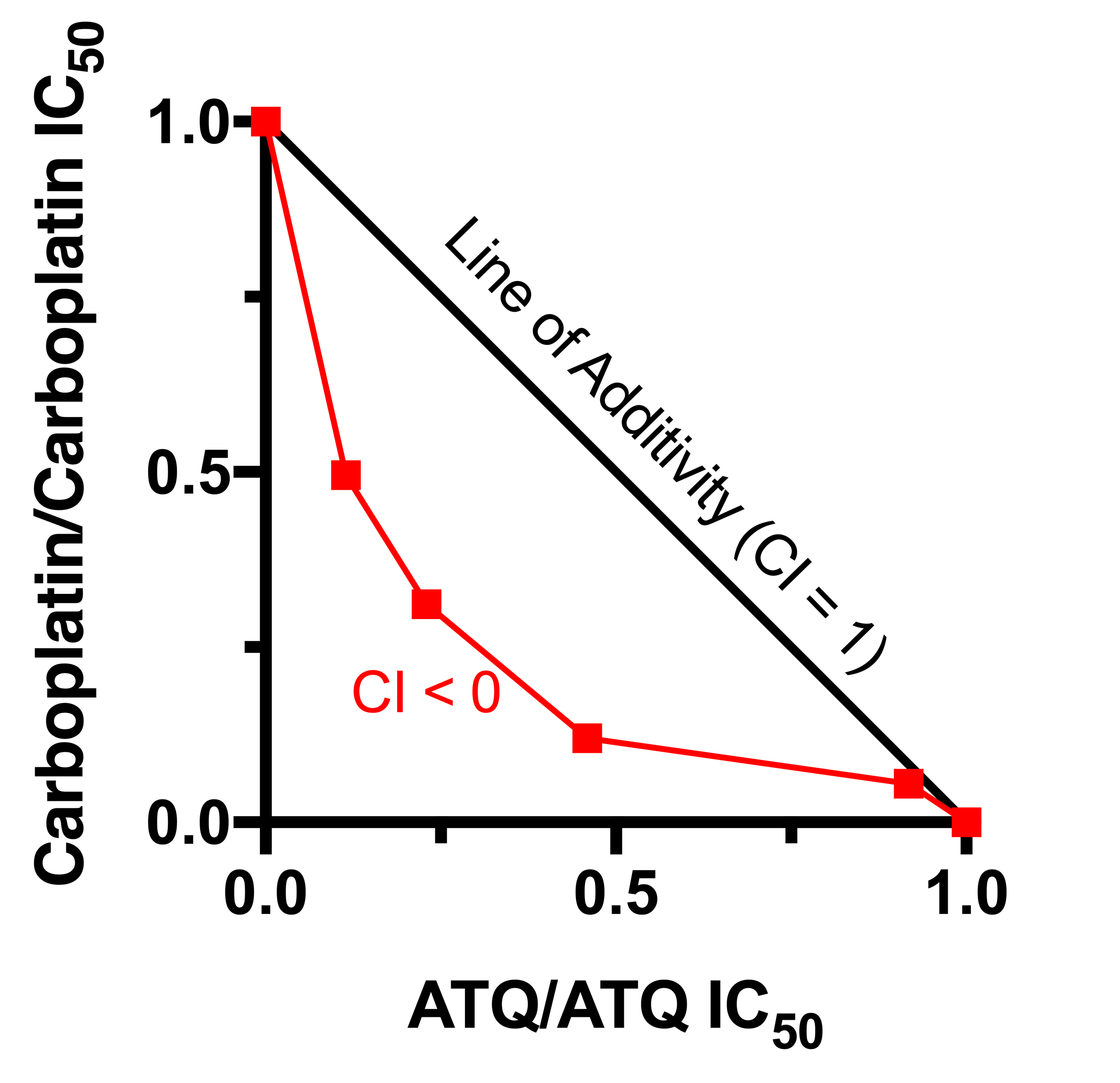

Supplement: Supplementary file 4 — Figure S3. Normalized Loewe’s Isobologram of ATQ in Combination with Carboplatin in vitro [file 41420_2020_343_MOESM4_ESM.tif]

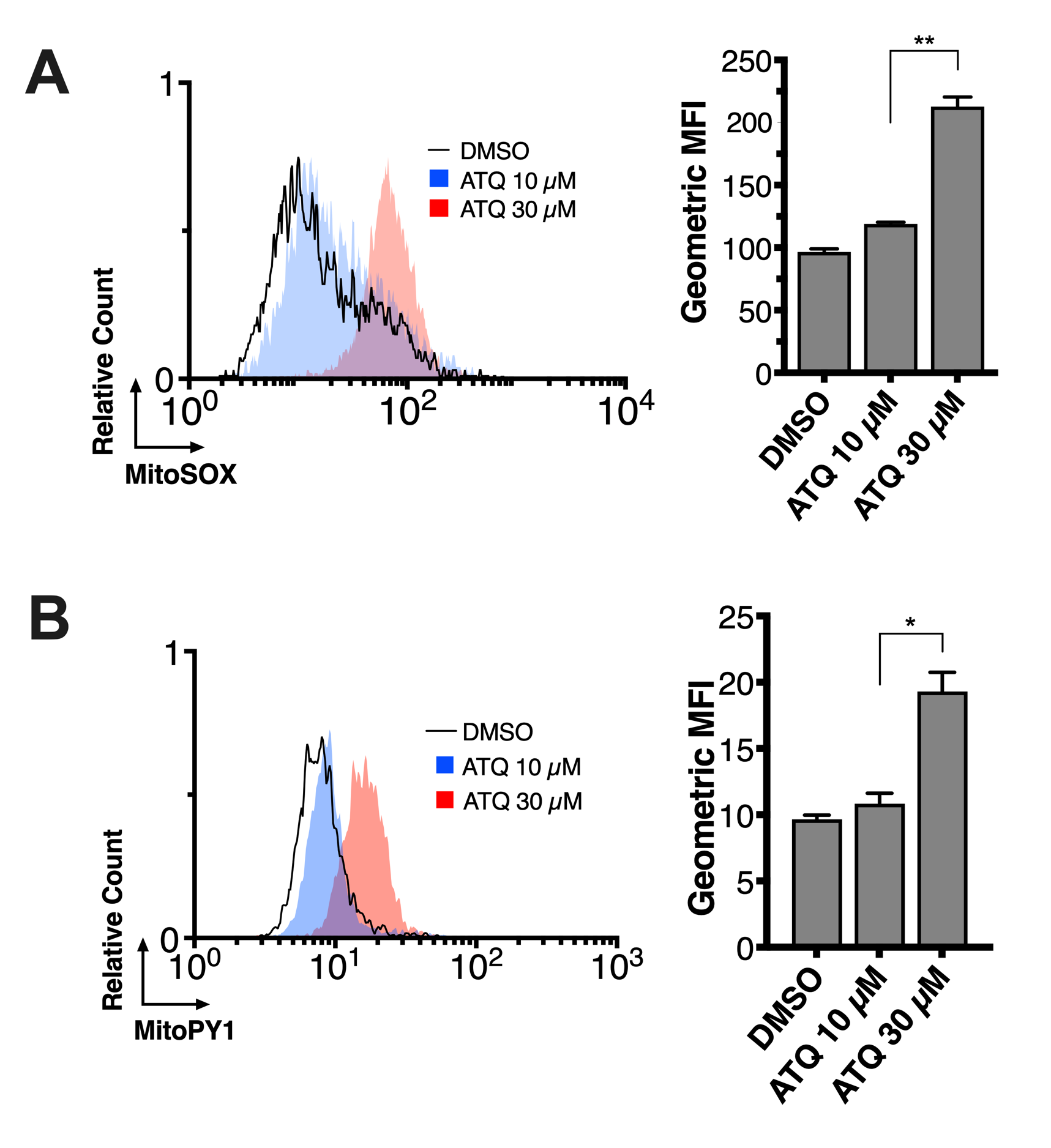

Supplement: Supplementary file 5 — Figure S4. ATQ Induces mROS in FaDu Cells [file 41420_2020_343_MOESM5_ESM.tif]

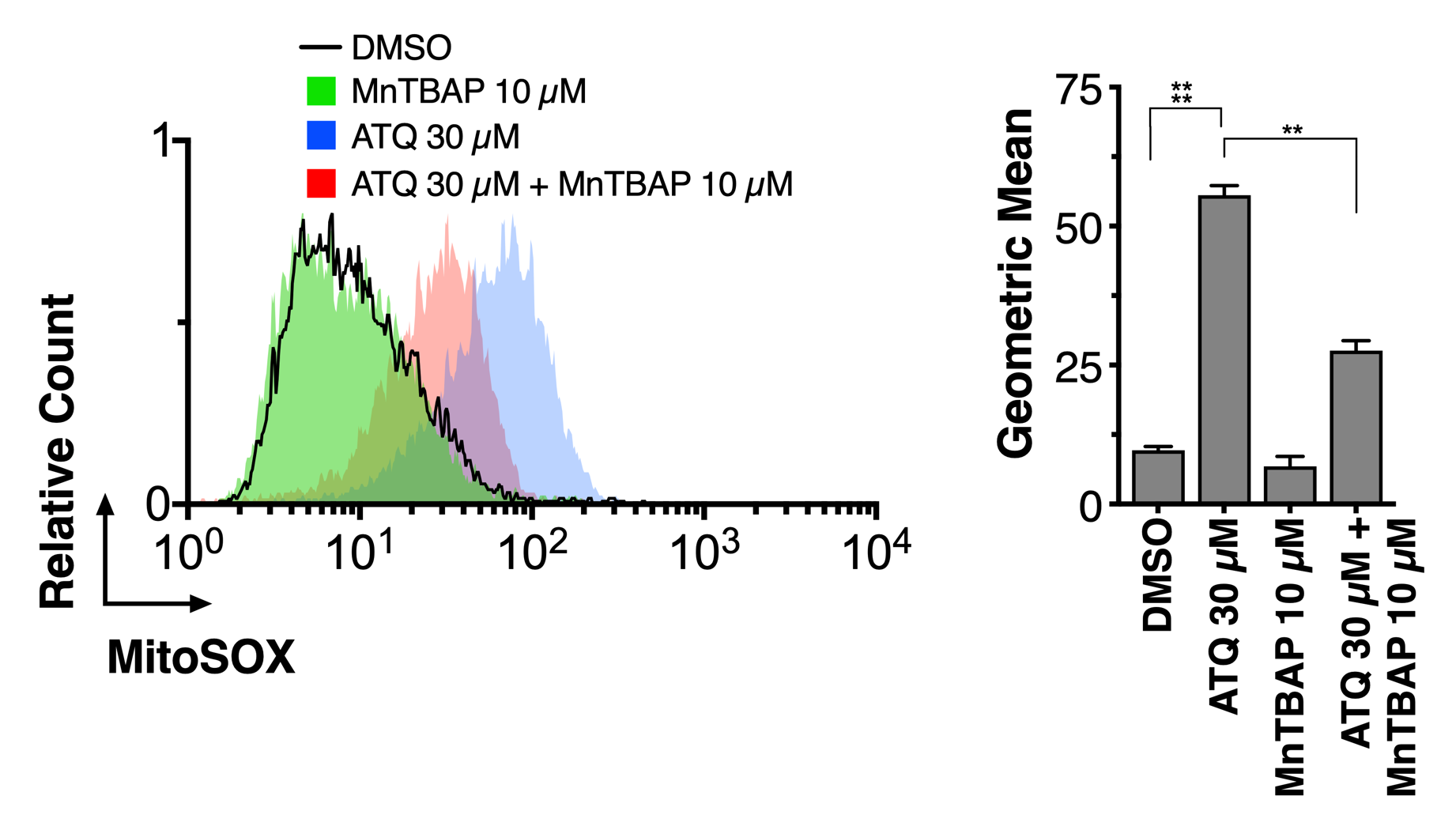

Supplement: Supplementary file 6 — Figure S5. MnTBAP Partially Abrogates ATQ-induced mROS in FaDu Cells [file 41420_2020_343_MOESM6_ESM.tif]

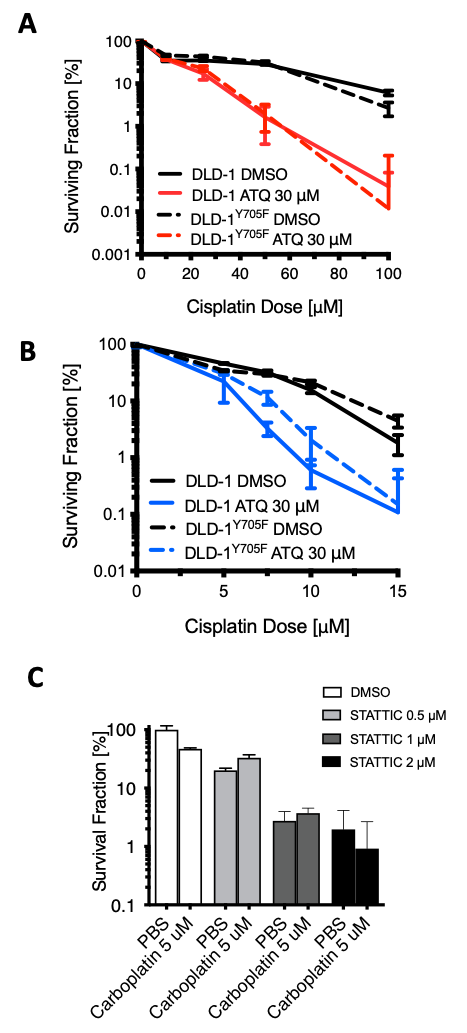

Supplement: Supplementary file 7 — Figure S6. Inhibition of pSTAT3Y705F does not Mediate ATQ-induced Platinum Sensitization [file 41420_2020_343_MOESM7_ESM.png]

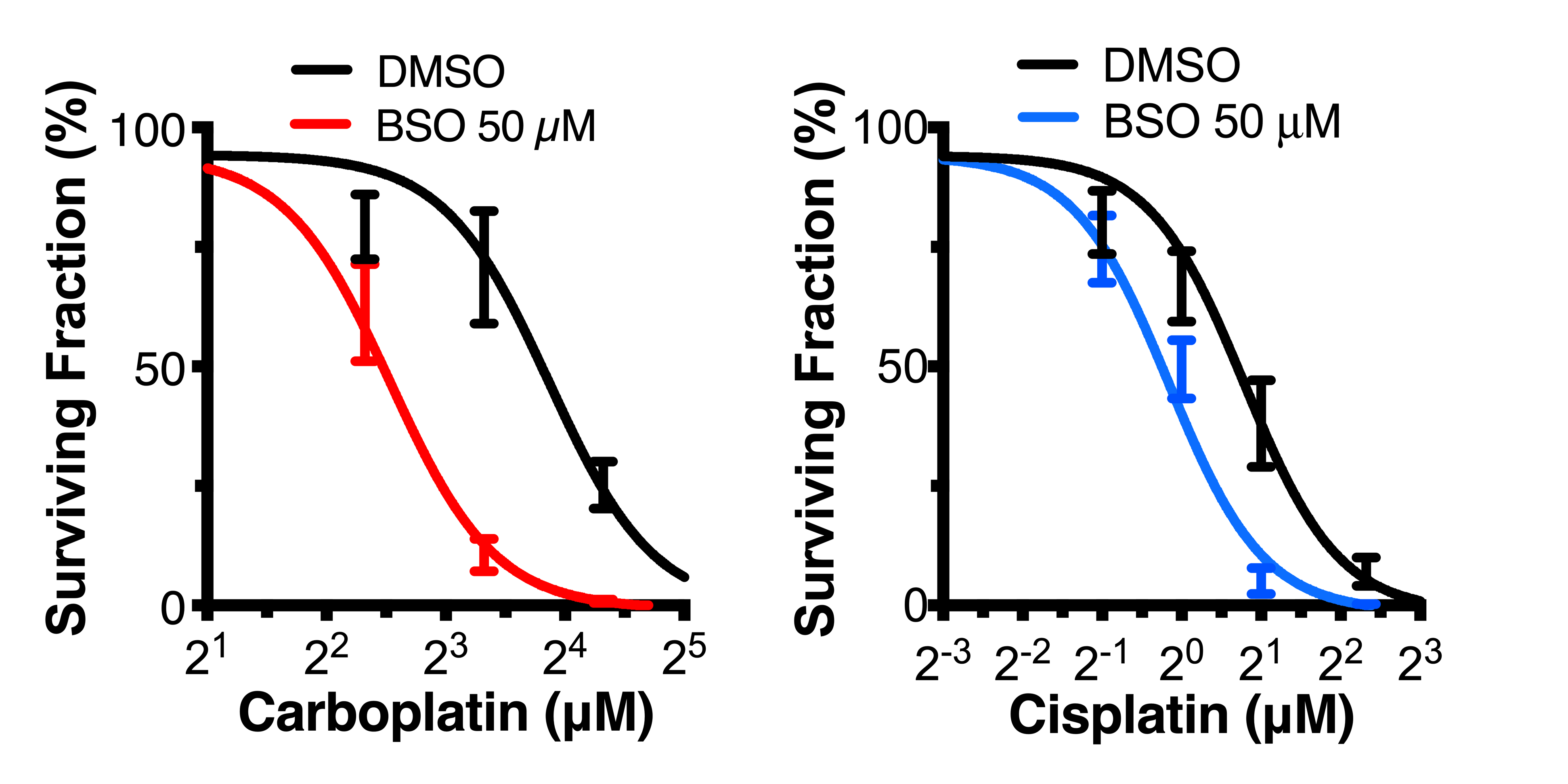

Supplement: Supplementary file 8 — Figure S7. BSO Sensitizes H460 Cells to Platinums in vitro [file 41420_2020_343_MOESM8_ESM.tif]

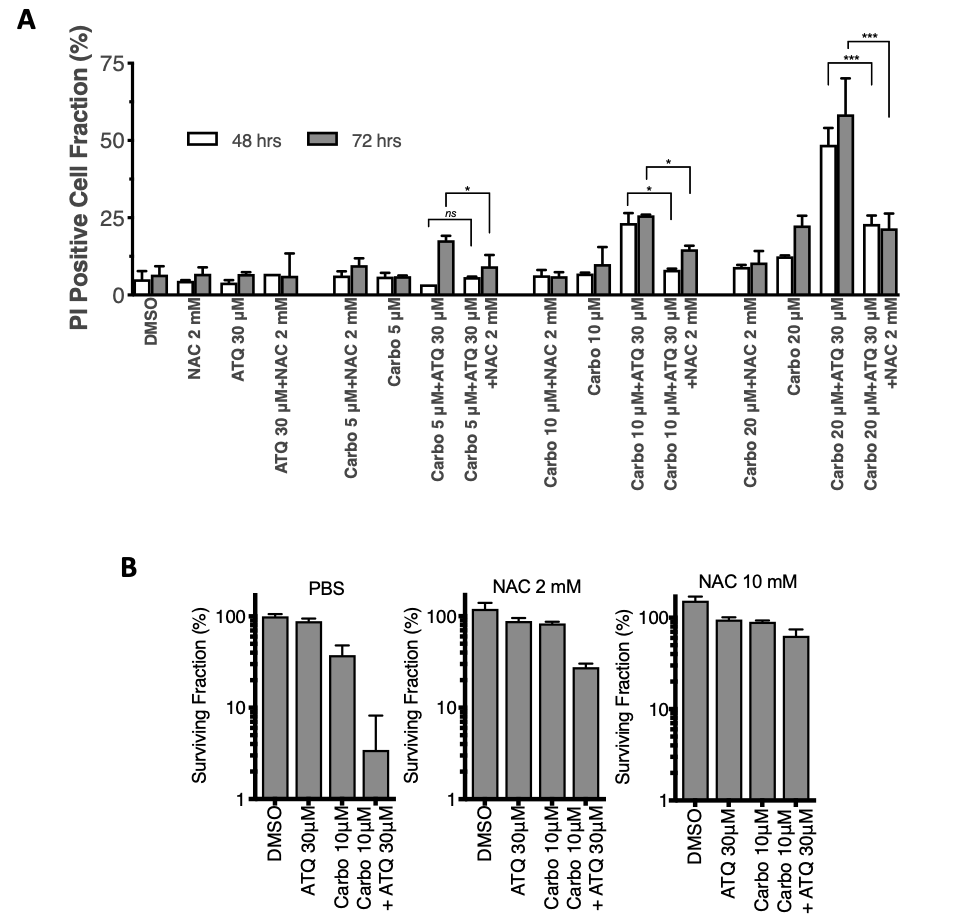

Supplement: Supplementary file 9 — Figure S8. NAC Rescues ATQ-induced Platinum Sensitization in FaDu Cells in vitro [file 41420_2020_343_MOESM9_ESM.png]
